# Single leg vertical jump performance identifies knee function deficits at return to sport after ACL reconstruction in male athletes

**DOI:** 10.1136/bjsports-2021-104692

**Published:** 2022-02-08

**Authors:** Argyro Kotsifaki, Sam Van Rossom, Rod Whiteley, Vasileios Korakakis, Roald Bahr, Vasileios Sideris, Ilse Jonkers

**Affiliations:** 1 Rehabilitation Department, Aspetar Orthopaedic and Sports Medicine Hospital, FIFA Medical Centre of Excellence, Doha, Qatar; 2 Department of Movement Sciences, KU Leuven Biomedical Sciences Group, Leuven, Belgium; 3 School of Human Movement & Nutrition Science, The University of Queensland, Saint Lucia, Queensland, Australia; 4 Department of Sports Medicine, Norwegian School of Sports Sciences, Oslo, Norway; 5 Aspetar Sports Injury and Illness Prevention Programme (ASPREV), Aspetar Orthopaedic and Sports Medicine Hospital, FIFA Medical Centre of Excellence, Doha, Qatar

**Keywords:** injury prevention, anterior cruciate ligament, knee injuries, sports

## Abstract

**Objectives:**

Vertical jump performance (height) is a more representative metric for knee function than horizontal hop performance (distance) in healthy individuals. It is not known what the biomechanical status of athletes after anterior cruciate ligament (ACL) reconstruction (ACLR) is at the time they are cleared to return to sport (RTS) or whether vertical performance metrics better evaluate knee function.

**Methods:**

Standard marker-based motion capture and electromyography (EMG) were collected from 26 male athletes cleared to RTS after ACLR and 22 control healthy subjects during single leg vertical jumps (SLJ) and single leg drop jumps (SLDJ). Performance outcomes, jump height and the Reactive Strength Index, were calculated. Sagittal plane kinematics, joint moments and joint work were obtained using inverse dynamics and lower limb muscle forces were computed using an EMG-constrained musculoskeletal model. Muscle contribution was calculated as a percentage of the impulse of all muscle forces in the model. Between-limb and between-group differences were explored using mixed models analyses.

**Results:**

Jump performance, assessed by jump height and Reactive Strength Index, was significantly lower in the involved than the uninvolved limb and controls, with large effect sizes. For the ACLR group, jump height limb symmetry index was 83% and 77% during the SLJ and SLDJ, respectively. Work generation was significantly less in the involved knee compared to uninvolved limb and controls during the SLJ (p<0.001; d=1.19; p=0.003, d=0.91, respectively) and during the SLDJ (p<0.001; d=1.54; p=0.002, d=1.05, respectively). Hamstrings muscle contribution was greater in the involved compared to the uninvolved limb and controls, whereas soleus contribution was lower in the involved limb compared to controls.

**Conclusions:**

During vertical jumps, male athletes after ACLR at RTS still exhibit knee biomechanical deficits, despite symmetry in horizontal functional performance and strength tests. Vertical performance metrics like jump height and RSI can better identify interlimb asymmetries than the more commonly used hop distance and should be included in the testing battery for the RTS.

**Key messages**

**What is already known on the topic?**

- At the time of return to sport, male athletes still present whole body biomechanical asymmetries, despite passing discharge criteria.

**What this study adds?**

- Anterior cruciate ligament reconstruction (ACLR) athletes achieved 97% symmetry in the horizontal performance (hop distance) but only and 83% and 77% symmetry in the vertical performance (jump height) of the single vertical jump and the single leg drop jump, respectively.

**How this study might affect research, practice or policy?**

- Our findings suggest that in a clinical setting jump height is a better metric than hop distance to evaluate the knee status at the time to return to sport after ACLR.
- Single leg drop jump performance metrics, like the jump height and reactive strength index, should be incorporated in the assessment of athletes after ACLR at the time to return to sport, when advanced equipment is not available

## Introduction

In professional male football players, 88% of anterior cruciate ligament (ACL) injuries occurred without direct knee contact.^1^ Usually, these injuries happen during single leg, decelerating change-of-direction activities, such as jump landing, cutting and pivoting.^2 3^

To reduce ACL reinjury incidence, a criteria-based rehabilitation and return to sport (RTS) progression has been proposed that is not solely governed by time after surgery.^4^ Typically, RTS testing relies on strength assessment and a battery of horizontal hop tests to assess functional symmetry between limbs.^5 6^ These functional tests have been widely adopted, as they are easy to administer and interpret without the need for expensive equipment. However, these tests are unable to predict successful RTS 1 year postinjury.^7^ Furthermore, evidence suggests that despite discharge criteria being reached, playing performance is still lower^8^ and reinjury rates remain high after RTS.^9^ A possible explanation is that the distance hopped is a poor measure of knee joint function; symmetry in hop distance performance does not ensure symmetry in lower limb biomechanics.^10^ Symmetry in hop distance is achieved earlier during rehabilitation than symmetry in isokinetic knee strength,^11 12^ suggesting that relying on hop distance Limb Symmetry Index (LSI) risks overestimating rehabilitation status.^13^

Tests that measure lower limb power output and the reactivity of an athlete might be more useful to assess the physical and performance readiness to RTS, like the vertical jumps. Current biomechanical tests used to screen healthy athletes for high-risk movement patterns in an attempt to identify risk for initial ACL injury, emphasise double-leg jumps, such as the drop vertical jump.^14–16^ Kinematics and kinetic variables were associated with future ACL injuries in an initial pilot study,^17^ however, this finding has not been replicated in larger studies.^18 19^ Although isolated double-leg sagittal plane tasks provide valuable information, they may not represent the injury risk in multidirectional sport athletes during more challenging single-leg movements.^20^

Accordingly, we aimed to describe in depth the biomechanical performance (kinematics, kinetics, work and individual muscle forces) of ACL-reconstructed athletes at the time they had met all the discharge criteria^6^ and compare them with healthy controls during propulsion and landing of a single leg vertical jump (SLJ) and during the reactive phase of a single leg drop jump (SLDJ). Our hypothesis was that athletes after ACL reconstruction (ACLR) would still display between-limb and between-group biomechanical differences, despite being cleared to RTS. Although these metrics provide detailed information on physical capacity, they require the use of sophisticated equipment, usually unavailable in the clinical setting. Thus, we aimed also to investigate if simpler vertical jumps performance metrics (jump height and Reactive Strength Index—RSI) can be used as surrogate measures for knee function of athletes after ACLR at the time to RTS.

## Methods

### Participants, inclusion and exclusion criteria

Forty-eight male participants were included in this study, 26 eligible patients after primary ACLR and 22 control subjects (table 1). Patients with ACLR were enrolled after the completion of a standardised rehabilitation protocol and after receiving clearance to RTS. The criteria for RTS were: (1) clearance by both their surgeon and physiotherapist, (2) completion of a sports-specific on-field rehabilitation programme, (3) quadriceps strength LSI >90% and (4) hop battery tests LSI >90%.^6^ ACLR patients were athletes (preinjury Tegner score ≥7) with a complete, unilateral ACL injury, either with an autologous ipsilateral bone-patellar-tendon-bone or a hamstrings graft (semitendinosus and gracilis), as clinically decided by the surgeon and athlete. Subjects with concomitant meniscal injuries that did not significantly impede the rehabilitation course were also included in the study. Potential participants were excluded if they had concomitant grade III knee ligament injury (other than ACL), full-thickness articular cartilage lesion, history of other lower extremity surgery (in either limb), back pain or lower extremity injury (other than primary ACL) in the 3 months prior to testing. Control subjects were athletes (Tegner score ≥7) recruited by contacting healthcare providers and sports club doctors. Inclusion criteria were: age range of 18–35 years, participation in level I or II sports three times a week or more, and no history of musculoskeletal injury of the lower limb 3 months prior to testing.

**Table 1:** Participant information

	ACLR group (n=26)	Control group (n=22)	P value
Age (years)	23.2±3.4	28.7±3.8	<0.001
Body mass (kg)	71.4±12.1	75.7±7.1	0.15
Height (cm)	173 (166–182)*	177.4±6.1	0.29
Body mass index (kg/m^2^)	23.3±2.3	24.0±1.6	0.23
Tegner score preinjury	9 (9–9)*	7 (7–9)*	<0.001
IKDC	94.9±7.0	100	0.002
ACL-RSI	92.0±10.6	NA	NA
Quadriceps strength LSI %	94±6	NA	NA
SLHD LSI %	97±4	100±5	0.011
TRHD LSI %	97±5	100 (98–102)*	0.07
Return to sport (months)	9.5±2.7	NA	NA
ACL hamstrings autograft, n (%)	10 (38)		
Isolated ACL injury, n	15		
Meniscal injury, n	11		
Cartilage lesion, n	2		

Values other than number of participants are expressed as mean±SD except where the data were non normally distributed where these data are presented as median and IQR.

Independent-sample t-tests were used for between groups comparison, significant difference (p<0.05).

*Non normally distributed data. All participants were male.

ACLR, anterior cruciate ligament reconstruction; ACL-RSI, Anterior Cruciate Ligament-Return to Sport after Injury scale; IKDC, International Knee Documentation Subjective Knee questionnaire; LSI, Limb Symmetry Index; NA, not available; SLHD, single leg hop for distance; TRHD, triple hop for distance.

Subjective knee function was evaluated using the International Knee Documentation Subjective Knee questionnaire^21^ and psychological readiness to RTS was measured by using ACL-RSI scale.^22^

### Equipment, participant preparation and marker set

Forty-two reflective markers were placed according to a full-body Plug-in-Gait marker-set, extended with additional anatomical markers on the sacrum, medial femur epicondyles and the medial malleoli.^23^ Three marker clusters replaced the single maker laterally on each thigh and shank since cluster-based models have less intersubject variance of frontal plane variables.^24^ The markers’ motion was captured with a 14-camera motion capture system (Vicon, Oxford, UK, 250 Hz). During the dynamic trials, ground reaction forces (GRFs) were collected synchronously with marker trajectories using five ground-embedded force plates (Kistler, Switzerland, 1000 Hz). Electromyography (EMG) activity was collected simultaneously with a 16-channel EMG system (Delsys Myomonitor IV, USA, 2000 Hz) from the vastus lateralis and medialis, rectus femoris, biceps femoris, semitendinosus medial and lateral gastrocnemius, and tensor fasciae latae (TFL) muscles of both limbs and in accordance with the surface EMG for the non-invasive assessment of muscle guidelines.^25^

### Experimental setup, procedure, and testing

**Figure 1:**
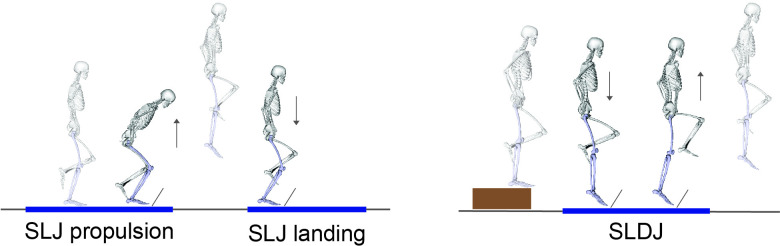
Visual representation of the tasks investigated. Single leg jump (SLJ) was analysed during the propulsion and the landing phase (left). Single leg drop jump (SLDJ) was analysed during the first (reactive) landing (right). For the SLDJ, work was calculated separately for the absorption phase (initial contact to peak knee flexion) and for the generation phase (peak knee flexion to toe-off).

All participants were evaluated at the same location by the same examiner and wore athletic shorts and standard shoes. They performed a 7 min warm up session including running, side running, deep squats and double leg jumps. Subsequently, they performed a series of maximal voluntary isometric contractions (MVIC) to obtain maximum EMG values for each measured muscle. The MVIC was assessed for TFL in standing, for quadriceps in sitting (60° of knee flexion), for hamstrings in prone (knee flexion at 30°) and for gastrocnemius in standing. A physiotherapist gave a verbal description and demonstrated the testing tasks. For SLJ, athletes started from an upright single leg standing position before briefly countermoving to a self-selected depth and then jumped vertically with maximum effort and landed on the same leg. Data were extracted for the two phases of the jump. The SLDJ was carried out from a 15 cm step. The subject was asked to drop from the step and on hitting the ground, immediately jump as high as possible while spending as little time as possible on the force plate. Only the first (reactive) landing was included for analysis. During both tasks, the subject’s hands were placed on their hips for consistency (figure 1). Data were collected bilaterally, and four successful trials were retained for analysis. Test limb order was simple randomised. Limb dominance was determined by asking the participants with which limb they would prefer to kick a ball.^26^

### Data processing

Data were processed in Visual 3D (C-Motion, Germantown, Maryland, USA). Marker trajectories and GRFs were low-pass filtered using a zero-lag, fourth order, Butterworth filter with the same 15 Hz cut-off frequency. For the SLJ, propulsion phase was defined as 0.4s prior to take off until take off—to include hip concentric phase which occurs before peak knee flexion, and landing phase from initial contact to peak knee flexion. Visual inspection of all trials was conducted to ensure that the whole concentric phase was included. For the SLDJ, we included in the analysis only the first (reactive) landing phase, defined from initial contact to toe off. Initial contact and toe off were expressed as the point when vertical GRF became less than 50N and more than 50N, respectively.

Joint angles were determined using a Visual 3D hybrid model with a Cardan X-Y-Z (mediolateral, anteroposterior, vertical) rotation sequence.^27^ Ankle, hip, and knee joint angles were defined as the angle between the distal and the proximal segment. Pelvis was defined using the Coda model.^28^ Pelvis and trunk segment angles were determined with respect to the global coordinate system. Joint internal moments were resolved in the proximal coordinate system. We calculated kinematics for trunk and pelvis, and also kinematics and kinetics for hip, knee and ankle joints, bilaterally. The variables of interest were performance metrics, joint angles, joint work and work contribution of each joint to the total work performed. Performance variables included jump height (measured as the vertical displacement of the centre of mass from toe off to the maximum height of the centre of mass), ground contact time and Reactive Strength Index (RSI). In the literature, RSI has been calculated using two methods: the jump height in a drop jump, divided by the contact time; or the flight time of the jump divided by the contact time, also reported as reactive strength ratio (RSR).^29 30^ We used both methods in our analysis. LSI was determined as the percentage of involved divided by uninvolved leg for the ACLR group and non-dominant leg divided by dominant leg for controls.^11 31^ Work generation was calculated as net joint power integrated over time in regions with positive internal power and work absorption in regions with negative internal power. The contribution of each joint was determined as a percentage of the sum of all three lower limb joints during each phase. Work and joint moments were normalised to body mass. Spatiotemporal, kinematic and kinetic variables were calculated bilaterally for hip, knee and ankle joints for both limbs.

### Estimation of muscle forces

We used a generic musculoskeletal model for deep squatting^32^ and followed a standard musculoskeletal modelling workflow implemented in Opensim 3.3^33^ to calculate muscle forces. The generic model was further optimised by including ligaments and opening additional degrees of freedom in the knee (ie, knee varus, valgus and knee internal-external rotation). Ligaments’ origin and insertion points described in the model of Xu *et al* 34 were registered on the Catelli model using host mesh fitting. We multiplied the maximum isometric force of each muscle by three to allow the generation of high forces required to perform the dynamic movements.^35 36^ The foot was modelled as one rigid segment. First, the generic model was scaled to the subject-anthropometrics and body mass. Subsequently, we used inverse kinematics to defer the joint kinematics from the measured marker trajectories, while internal joint moments were determined using inverse dynamics implemented in Opensim. An EMG-constrained static optimisation approach that omitted the force-length and force-velocity behaviour^37^ was used to determine the muscle forces required to balance the external joint moments. For this, EMG signals were first filtered with a fourth order Butterworth bandpass filter with 20–400 Hz filter thresholds, then rectified and filtered with a second order low-pass Butterworth filter at 10 Hz, and finally, normalised to the peak EMG value measured for the subject across all activities performed during data collection, which included maximum voluntary contractions, running, jumping, cutting and hopping for maximum distance.^38^ To account for participant-specific mass, muscle forces were normalised to body weight. Finally, we calculated the relative contribution of each muscle force impulse during each task as a percentage of the impulse of all muscle forces (30 in total for each leg) in the model.

### Statistical analysis

Descriptive statistics were used to summarise the characteristics of the participants and measurements. Normality of distribution of data was assessed with the Shapiro-Wilk test^39^ and Q-Q plots. For the analysis we used only one (randomly selected) control limb from each control subject. Between-limbs (involved, uninvolved and control) comparisons were assessed using mixed-effect models with subject-specific random effects. Post hoc comparisons (Tukey) were performed to adjust for multiple comparisons. The parameter estimates were adjusted for age, Tegner score and body mass index. A p<0.05 was considered for statistical significance. Effect sizes (ESs) were calculated using the pooled^40^ (between-limb) and the pooled weighted^41^ (between-group) SD. Values of 0.2, 0.5 and 0.8 were identified as the lower thresholds for small, moderate and large effects, respectively.^40^ All statistical analysis was performed using JMP V.15 (SAS Institute).

## Results

Participants were tested within 2 weeks of clearance to RTS, at 9.5±2.7 months following ACLR. Jump performance in both tests, assessed by jump height and RSI, was significantly lower in the involved than the uninvolved limb and controls with large ES (tables 2 and 3).

**Table 2:** Comparison between groups during the propulsive and landing phases of the single leg vertical jump

	Involved	Uninvolved	Controls	Involved-uninvolved			Involved controls			Uninvolved controls		
				P value	d	Mean difference (95% CIs)	P value	d	Mean difference (95% CIs)	P value	d	Mean difference (95% CIs)
Jump height (cm)	11.0±3.5	13.3±3.3	13.7±2.1	**<0.001**	**0.67**	−2.3 (−3.4 to −1.2)	**0.016**	**0.87**	−2.4 (−4.5 to −0.4)	0.98		−0.2 (−2.2 to 1.9)
**Propulsion**												
Peak joint motion (**°**)												
Hip flexion	82.9±12.84	78.6±12.1	85.3±12.8	**0.003**	**0.34**	4.3 (1.3 to 7.3)	0.15		−8.1 (−18.4 to 2.3)	**0.016**	**0.53**	−12.4 (−22.7 to −2.0)
Knee flexion	70.4±7.40	74.0±5.7	75.1±5.9	**0.007**	**0.54**	−3.6 (−6.3 to −0.9)	**0.043**	**0.67**	−4.7 (−9.2 to −0.1)	0.83		−1.1 (−5.6 to 3.5)
Ankle dorsiflexion	29.6±3.47	31.4±2.81	31.0±4.0	**0.022**	**0.58**	−1.8 (−3.4 to −0.2)	0.37		−1.4 (−3.9 to 1.1)	0.91		0.4 (−2.1 to 2.9)
Trunk flexion	49.1±11.85	43.3±12.64	47.1±13.3	**<0.001**	**0.48**	5.5 (2.9 to 8.2)	0.68		3.4 (−6.4 to 13.1)	0.86		−2.1 (−11.9 to 7.6)
Anterior pelvic tilt	44.6±9.92	39.5±8.90	44.6±10.1	**<0.001**	**0.54**	5.1 (2.7 to 7.5)	1.00		−0.0 (−6.9 to 6.9)	0.19		−5.1 (−12.0 to 1.9)
Joint moments (Nm/kg)												
Hip flexion	−3.3±0.5	−3.0±0.7	−3.0±0.6	0.05		−0.2 (−0.5 to 0.0)	0.46		−0.2 (−0.7 to 0.2)	1.00		0.02 (−0.4 to 0.5)
Knee flexion	−1.9±0.3	−2.3±0.4	−2.1±0.4	**<0.001**	**1.23**	0.4 (0.3 to 0.6)	0.38		0.1 (−0.1 to 0.4)	**0.019**	**0.77**	−0.3 (−0.5 to 0.04)
Ankle dorsiflexion	−2.3±0.3	−2.3±0.3	−2.2±0.2	0.51		0.1 (−0.1 to 0.2)	0.42		−0.1 (−0.3 to 0.1)	0.13		−0.2 (−0.4 to 0.04)
Work (J/kg)												
Hip	1.5±0.6	1.4±0.6	1.4±0.3	0.64		0.1 (−0.1 to 0.3)	0.93		0.1 (−0.3 to 0.4)	1.00		−0.01 (−0.4 to 0.3)
Knee	1.2±0.4	1.6±0.3	1.5±0.4	**<0.001**	**1.19**	−0.4 (−0.6 to −0.2)	**0.003**	**0.91**	−0.3 (−0.6 to −0.1)	0.86		0.1 (−0.2 to 0.3)
Ankle	1.4±0.4	1.6±0.3	1.5±0.3	**0.002**	**0.62**	−0.2 (−0.4 to −0.1)	0.81		−0.1 (−0.3 to 0.2)	0.28		0.2 (−0.1 to 0.4)
Total	4.1±0.9	4.6±0.8	4.4±0.6	**<0.001**	**0.59**	−0.5 (−0.8 to −0.2)	0.33		−0.3 (−0.4 to 0.8)	0.72		0.2 (−0.4 to 0.8)
**Landing**												
Peak joint motion (**°**)												
Hip flexion	67.3±16.8	62.2±14.8	64.7±19.1	**0.003**	**0.32**	5.1 (1.6 to 8.6)	0.95		−1.8 (−16.6 to 13.1)	0.51		−6.8 (−21.7 to 8.0)
Knee flexion	61.1±11.2	64.8±10.3	62.1±10.2	**0.001**	**0.34**	−4.0 (−6.6 to −1.5)	0.91		−1.6 (−10.8 to 7.7)	0.79		2.5 (−6.8 to 11.7)
Ankle dorsiflexion	26.0±4.2	28.0±3.3	27.2±4.6	**0.003**	**0.51**	−2.1 (−3.5 to −0.6)	0.74		−1.1 (−4.7 to 2.5)	0.79		1.0 (−2.6 to 4.6)
Trunk flexion	32.5±13.3	28.5±15.9	26.4±11.7	**0.018**	**0.27**	4.0 (0.6 to 7.4)	0.91		2.0 (−9.5 to 13.4)	0.90		−2.1 (−13.5 to 9.4)
Anterior pelvic tilt	34.7±10.0	29.1±8.3	33.9±12.0	**<0.001**	**0.62**	5.7 (2.7 to 8.6)	0.96		0.8 (−6.6 to 8.3)	0.27		−4.8 (−12.3 to 2.6)
Joint moments (Nm/kg)												
Hip flexion	−3.6±1.4	−3.3±1.5	−2.8±1.4	0.26		−0.2 (−0.6 to 0.1)	0.20		−0.7 (−1.7 to 0.3)	0.48		−0.5 to −1.5 to 0.5)
Knee flexion	−2.4±0.6	−2.9±0.5	−2.7±0.6	**<0.001**	**0.85**	0.4 (0.3 to 0.6)	0.31		0.3 (−0.2 to 0.7)	0.51		−0.2 (−0.6 to −0.2)
Ankle dorsiflexion	−2.5±0.4	−2.6±0.5	−2.4±0.4	0.16		0.2 (−0.03 to 0.3)	0.77		−0.1 (−0.4 to 0.2)	0.27		−0.2 (−0.5 to 0.1)
Work (J/kg)												
Hip	−1.2±0.5	−1.1±0.4	−1.1±0.5	0.79		−0.04 (−0.2 to 0.1)	0.83		−0.1 (−0.4 to 0.3)	0.96		−0.04 (−0.4 to 0.3)
Knee	−0.9±0.4	−1.1±0.4	−1.3±0.5	**0.014**	**0.52**	0.2 (0.04 to 0.4)	**0.049**	**0.69**	0.3 (0.0 to 0.6)	0.68		0.1 (−0.2 to 0.4)
Ankle	−1.0±0.4	−1.1±0.4	−1.2±0.4	**0.038**	**0.39**	0.2 (0.01 to 0.3)	0.14		0.2 (−0.1 to 0.5)	0.85		0.1 (−0.2 to 0.3)
Total	−3.1±0.7	−3.4±0.6	−3.5±0.7	**<0.001**	**0.49**	0.3 (0.2 to 0.5)	0.07		0.5 (−0.03 to 1.0)	0.78		0.1 (−0.4 to 0.6)

For the ACLR athletes, data are presented for both the reconstructed (‘involved’) and contralateral (‘uninvolved’) leg along with one (randomly selected) leg of the control subjects (‘controls’) as mean±SD The between-group comparisons show p values, non-standarised mean differences and 95% CIs and effect sizes (d) only where p<0.05. Bold indicates statistically significant differences and their respective effect sizes.

ACLR, anterior cruciate ligament reconstruction.

**Table 3:** Comparison between groups during the propulsive and landing phases of the single leg drop jump

	Involved	Uninvolved	Controls	Involved-uninvolved			Involved-controls			Uninvolved-controls		
				P value	d	Mean difference (95% CIs)	P value	d	Mean difference (95% CIs)	P value	d	Mean difference (95% CIs)
Jump height (cm)	10.6±3.4	13.8±3.5	14.4±3.1	**<0.001**	**0.92**	−3.2 (−4.0 to −2.3)	**0.001**	**1.12**	−3.7 (−6.1 to −1.4)	0.84		−0.6 (−2.9 to 1.8)
RSI (jump height/contact time) (cm/s)	0.30±0.10	0.43±0.12	0.43±0.12	**<0.001**	**1.18**	−0.1 (−0.2 to −0.1)	**0.001**	**1.16**	−0.1 (−0.2 to −0.1)	0.98		−0.01 (−0.1 to 0.1)
RSR (flight time/contact time)	0.93±0.17	1.10±0.19	1.09±0.24	**<0.001**	**0.94**	−0.2 (−0.2 to 0.1)	**0.042**	**0.76**	−0.2 (−0.3 to −0.0)	0.98		0.01 (−0.1 to 0.2)
Contact time (s)	0.36±0.05	0.33±0.05	0.35±0.06	**<0.001**	**0.60**	0.02 (0.01 to 0.04)	0.79		0.01 (−0.03 to 0.1)	0.64		−0.01 (−0.1 to 0.02)
Peak joint motion (°)												
Hip flexion	61.5±12.5	54.1±10.8	54.0±11.6	**<0.001**	**0.63**	7.4 (4.3 to 10.5)	0.09		7.5 (−0.9 to 15.8)	1.00		0.1 (−8.3 to 8.4)
Knee flexion	59.0±7.9	61.8±6.7	60.5±7.5	**0.026**	**0.38**	−2.8 (−5.3 to −0.3)	0.79		−1.5 (−6.8 to 3.9)	0.81		1.4 (−4.0 to 6.7)
Ankle dorsiflexion	22.9±3.1	24.4±2.4	23.9±4.0	**0.001**	**0.54**	−1.5 (−2.7 to −0.3)	0.88		−0.5 (−3.0 to 2.0)	0.60		1.0 (−1.5 to 3.5)
Trunk flexion	27.8±8.4	20.7±8.1	21.1±9.1	**<0.001**	**0.87**	7.1 (4.9 to 9.4)	**0.031**	**0.75**	6.7 (0.5 to 12.9)	0.98		−0.4 (−6.6 to 5.8)
Anterior pelvic tilt	30.9±7.8	24.4±6.8	25.0±7.5	**<0.001**	**0.89**	6.5 (4.1 to 8.9)	**0.028**	**0.75**	5.9 (0.6 to 11.2)	0.95		−0.6 (−5.9 to 4.7)
Joint moments (Nm/kg)												
Hip flexion	−3.7±0.5	−3.1±0.6	−3.2±0.6	**<0.001**	**0.95**	−0.5 (−0.8 to −0.2)	**0.028**	**0.81**	−0.5 (−0.9 to −0.04)	0.94		0.1 (−0.4 to 0.5)
Knee flexion	−3.0±0.5	−3.8±0.6	−3.2±0.6	**<0.001**	**1.44**	0.8 (0.5 to 1.1)	0.21		0.3 (−0.1 to −0.7)	**0.008**	**0.88**	−0.5 (−0.9 to 0.1)
Ankle dorsiflexion	−2.8±0.5	−2.9±0.5	−2.9±0.5			0.2 (−0.01 to 0.3)	0.73		0.1 (−0.3 to 0.5)	0.93		−0.1 (−0.4 to 0.3)
Work absorption (J/kg)												
Hip	−0.9±0.3	−0.7±0.2	−0.7±0.2	**0.003**	**0.84**	−0.2 (−0.4 to −0.1)	**0.017**	**0.86**	−0.2 (−0.4 to −0.03)	0.97		0.02 (−0.2 to 0.2)
Knee	−0.9±0.3	−1.1±0.3	−1.1±0.3	**<0.001**	**0.80**	0.2 (0.1 to 0.3)	0.21		0.2 (−0.1 to 0.4)	0.79		−0.1 (−0.3 to 0.2)
Ankle	−0.9±0.2	−1.1±0.3	−1.2±0.4	**0.022**	**0.56**	0.1 (0.02 to 0.3)	**0.014**	**0.85**	0.3 (0.1 to 0.6)	0.26		0.2 (−0.1 to 0.4)
Total	−2.7±0.5	−2.9±0.4	−3.0±0.5	0.16		0.1 (−0.04 to 0.3)	0.17		0.3 (−0.1 to 0.6)	0.68		0.1 (−0.2 to 0.5)
Work generation (J/kg)												
Hip	1.1±0.3	0.8±0.3	0.8±0.2	**<0.001**	**0.87**	0.2 (0.1 to 0.4)	**0.015**	**0.95**	0.2 (0.04 to 0.4)	0.96		−0.02 (−0.2 to 0.2)
Knee	1.0±0.3	1.5±0.3	1.3±0.3	**<0.001**	**1.54**	−0.4 (−0.6 to −0.3)	**0.002**	**1.05**	−0.3 (−0.5 to −0.1)	0.24		0.1 (−0.1 to 0.3)
Ankle	1.4±0.4	1.5±0.3	1.5±0.4	**<0.001**	**0.50**	−0.2 (−0.3 to −0.1)	0.25		−0.2 (−0.4 to 0.1)	1.00		0.0 (−0.3 to 0.3)
Total	3.4±0.6	3.8±0.6	3.7±0.5	**<0.001**	**0.59**	−0.4 (−0.6 to −0.2)	0.31		0.3 (−0.7 to 0.2)	0.80		0.1 (−0.3 to 0.5)

For the ACLR athletes, data are presented for both the reconstructed (‘involved’) and contralateral (‘uninvolved’) leg along with one (randomly selected) leg of the control subjects (‘controls’) as mean±SD. The between group comparisons show p values, non-standarised mean differences and 95% CIs and effect sizes (d) only where p<0.05. Bold indicates statistically significant differences and their respective effect sizes.

ACLR, anterior cruciate ligament reconstruction; RSI, Reactive Strength Index; RSR, reactive strength ratio.

The following biomechanical differences between limbs and between groups were identified:

Kinematics. Sagittal plane kinematic differences were found during propulsion and landing of the SLJ and the SLDJ, with more peak hip flexion, ankle plantarflexion, trunk flexion and anterior pelvis tilt, but less peak knee flexion of the involved compared with the uninvolved limb (tables 2 and 3).

Knee flexion moments. Knee flexion moments were lower in the involved limb compared with the uninvolved during propulsion (p<0.001, d=1.23) and landing (p<0.001, d=0.85) of the SLJ and during the SLDJ (p<0.001, d=1.44) (tables 2 and 3).

Work. During SLJ, knee work in the involved limb was lower that the uninvolved and controls in both propulsion and landing phases. Ankle work was lower in the involved limb than the uninvolved in both propulsion and landing, with no difference limb the control group. Hip work had no difference between limbs or between groups. Total lower limb work was lower in the involved limb compared with the uninvolved limb in both propulsion and landing. During SLDJ, knee and ankle work was lower in the involved than the uninvolved limb in both absorption and generation phases, while hip work was greater in the involved compared with the uninvolved and controls in both absorption and generation phases. Total lower limb work was lower in the involved limb compared with the uninvolved limb during absorption but not during the generation phase (tables 2 and 3).

**Figure 2:**
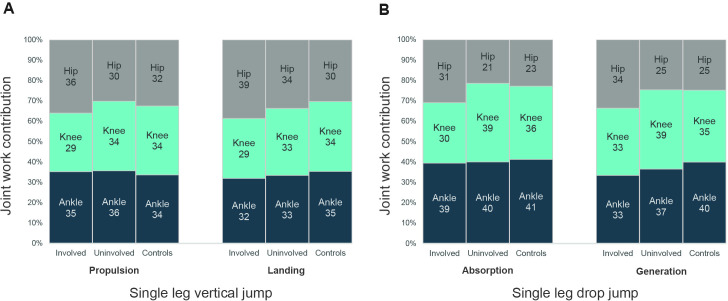
Average percentage work contributions from the hip, knee and ankle joints during the propulsion and landing phases of the single leg vertical jump (A) and the absorption and generation phase of the first landing (reactive landing) of the single leg drop jump (B).

Work contribution. During propulsion of the SLJ, work contribution was less in the involved knee and more in the involved hip compared with the uninvolved limb. No differences were observed during landing (figure 2A). During the absorption phase of the SLDJ, work contribution was lower in the involved knee and greater in the involved hip compared with the uninvolved limb. During generation (push-off phase), work contribution was less in the involved knee and more in the involved hip compared with the uninvolved limb and controls with large ES (figure 2B, online supplemental file 1).

10.1136/bjsports-2021-104692.supp1 Supplementary data

**Figure 3:**
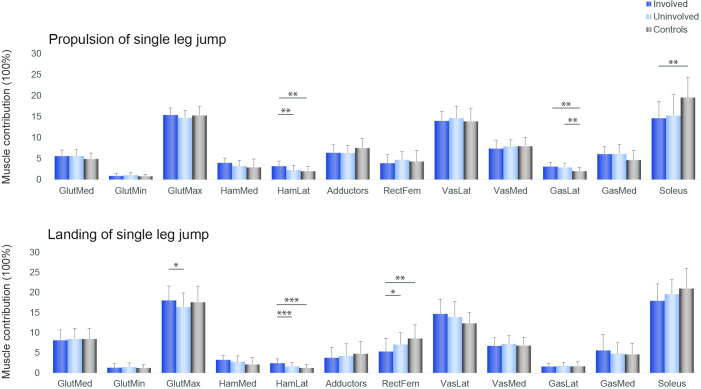
Individual muscle forces impulse contributions for the involved leg (dark blue), the uninvolved (light blue) and the controls (grey), during the propulsive (top) and landing phases (bottom) of the single leg vertical jump *p<0.05, **p<0.01, ***p<0.001. From left to right: gluteus medius, gluteus minimus, gluteus maximus, medial hamstrings, lateral hamstrings, hip adductors, rectus femoris, vastus lateralis, vastus medialis, gastrocnemius lateral head, gastrocnemius medial head, soleus.

**Figure 4:**
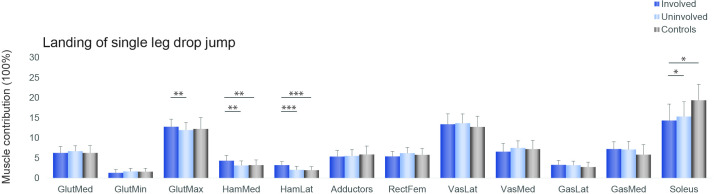
Individual muscle forces impulse contributions for the involved leg (dark blue), the uninvolved (light blue) and the controls (grey), during the first landing (reactive landing) of the single leg drop jump *p<0.05, **p<0.01, ***p<0.001. From left to right: gluteus medius, gluteus minimus, gluteus maximus, medial hamstrings, lateral hamstrings, hip adductors, rectus femoris, vastus lateralis, vastus medialis, gastrocnemius lateral head, gastrocnemius medial head, soleus.

**Figure 5:**
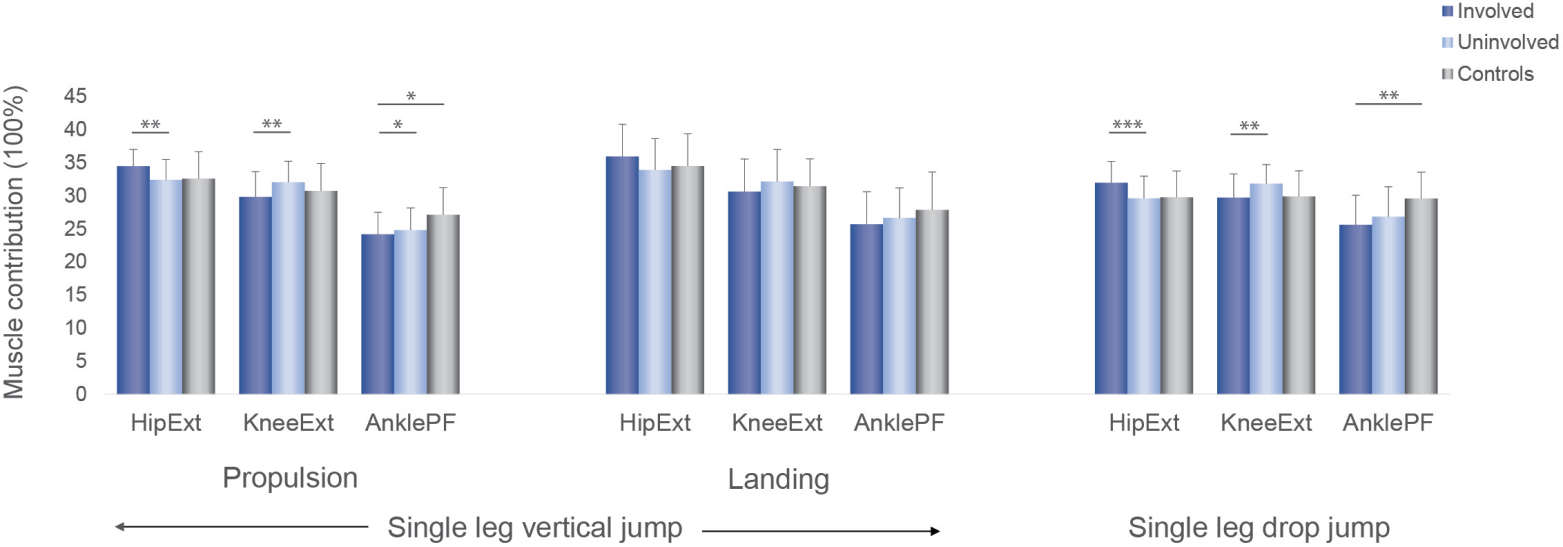
Muscle contributions (%) during the propulsive and landing phases of the single leg vertical jump and during first landing of the single leg drop jump for the involved leg (dark blue), the uninvolved (light blue), and the controls (grey), *p<0.05, **p<0.01, ***p<0.001. AnklePF, ankle plantar-flexors; HipExt, hip extensors; KneeExt, knee extensors.

Muscle forces. Hamstrings muscle contribution was greater in the involved compared with the uninvolved limb and controls, while soleus contribution was bilaterally lower in the ACLR group compared with controls (figures 3–5).

## Discussion

### Performance metrics

Despite passing the recommended test criteria to RTS after ACLR, significant asymmetries in vertical jump performance were identified in male athletes, as well as differences compared with a healthy comparison group. In addition to the performance differences, differences in knee work, kinematics and muscle function were also evident.

In a previous paper using the same patient and control population, Kotsifaki *et al*, documented that athletes after ACLR displayed knee function deficits despite achieving almost symmetrical hop distance (97% LSI).^10^ However, knee function deficits are evident in vertical performance criteria (jump height), with 83% and 77% symmetry for the jump height during the SLJ and SLDJ, respectively. In the control group the symmetry in jump height was 98% and 100% during the SLJ and SLDJ, respectively. Evaluating the knee work differences between limbs as LSI, knee work generation during the SLJ was 75% and during the SLDJ was 70%. In the control group, knee work generation symmetry was 100% and 99% during the SLJ and the SLDJ, respectively.

Vertical and horizontal hops measure different aspects of lower limb function. Jump performance (jump height) is mostly related to the concentric phase of a vertical jump task and assesses the ability to take off with powerful extension of the hip, knee, and ankle.^42^ In healthy individuals, the relative knee work contribution in the concentric phase was three times greater during the vertical hop than during the horizontal hop.^43^ In the same cohort as the current study, the knee contribution to the total lower limb work in the concentric phase of a horizontal hop was only 10%.^10^ However, during the concentric phase of both the SLJ and the SLDJ, knee contribution was approximately 30% of the total work. The greater knee work contribution during vertical hops likely explains why performance deficits are more readily apparent than during horizontal hops in those with knee impairments. Accordingly, we suggest that vertical hop performance (height) is a better metric than horizontal hop distance to assess knee function.

Previous research has also reported that symmetry in vertical jump height was more difficult to achieve than symmetry in horizontal hop distance.^44–47^ Additionally, symmetry in hop distance is achieved earlier during rehabilitation than symmetry in isokinetic knee strength^11 12^ suggesting that emphasising hop distance symmetry risks overestimating rehabilitation status.^45 48^ In contrast, vertical jump performance metrics remain impaired, even when athletes have passed all our discharge criteria.^6^

Jump height and RSI have been previously used to quantify drop-jump performance. The current data show greater knee work deficits during SLDJ (70% LSI) than SLJ (75% LSI) which suggests the addition of the reactive phase may better highlight deficits in knee function. Calculating knee work is typically not possible in clinical situations where biomechanical analyses are not available, however, estimating jump height and RSI is more feasible for clinicians (contact mats, phone-based apps, photoelectronic cells, etc).

### Whole-body compensations

In both jumping tasks, athletes after ACLR display a more extended knee position and more hip flexion, anterior pelvic tilt, and trunk flexion. Additionally, during the SLDJ and the propulsion phase of the SLJ, athletes after ACLR displayed less knee work contribution of the involved limb but compensated with more hip work compared with the uninvolved limb. This might represent a mechanism to compensate for reduced knee work found in all phases of both tasks. The early part of the landing phase is when ACL injuries happen^3^ and patients after ACLR has been shown to shift the demands away from the involved knee. A less flexed knee at landing is potentially more vulnerable to ACL reinjury suggesting this compensatory strategy may heighten reinjury risk. The utilisation of intralimb compensation to offload the knee and overload the hip is a mechanism commonly seen in various tasks after ACLR,^49–51^ and is also influenced by gender and strength, with men shifting the energy absorption mainly to the hip.^52^ Furthermore, the upper body movement pattern was different when the task was performed with the involved as compared with the uninvolved limb. This suggests that this compensatory strategy extends beyond the lower limb, to the whole body.

### Muscle contribution

Patients after ACLR displayed asymmetries in relative muscle contribution, indicating altered muscle coordination strategies. In both vertical jumps, the lateral hamstrings contribution was significantly greater in the involved limb than the uninvolved and the control group. Increased hamstrings activation is a commonly reported strategy in patients with ACL deficiency^53 54^ and in subjects after ACLR.^55 56^ Increased co-contraction of the hamstring muscles is considered protective, as the hamstrings have a posterior line of pull in a flexed knee and might thus act as an ACL agonist, counteracting high anterior tibial shear forces.^57^ A neuromuscular modelling approach indicated that during single leg drop landing, the muscles that generated the greatest ACL-protective posterior shear force, were the soleus, medial hamstrings and biceps femoris.^58^ In our cohort of ACLR patients although, the contribution of the medial and lateral hamstrings in the involved limb was greater than the uninvolved, but soleus contribution was less than controls bilaterally. In the control group, the ankle appears to contribute 5% more and the hip less during the SLDJ compared with the SLJ. Similarly, there is more muscle contribution of ankle plantar flexors and less from the hip extensors during the SLDJ compared with the SLJ (figure 5). Performance of the SLDJ is largely affected not only by the status of the knee but also the status of the ankle. This may explain the larger asymmetries observed in the performance metrics of the SLDJ compared with the SLJ, given the lower contribution of soleus in our cohort.

### Clinical implications

Measuring vertical jump performance (height) is a better tool to evaluate knee function than the more commonly performed horizontal hop outcomes (distance) in ACLR patients. In a clinical setting or in the field, where no access to advanced equipment is available, we recommend the use of the vertical jump height symmetry as a discharge criterion instead of the currently used hop distance symmetry as this better represents the knee status of an athlete.

Three-dimensional biomechanical measures are used as the gold standard for assessing an athlete’s movement quality and performance after ACLR and provide significantly more information on physical capacities. These methods, however, are largely unavailable in the clinical setting. Recent improvements in technology allow the use for valid and reliable alternative methods to measure vertical jump performance, such as low-cost force plates, contact mats, photoelectric cells or even mobile applications.^59–64^ Jump height and RSI may be better markers of rehabilitation progression than the more commonly used hop distance.

The observed reduction in soleus work during the landing and propulsion phases suggests that rehabilitation strategies to increase soleus strength may be indicated as a means of reducing ACL loading.

### Methodological considerations

These data cannot be generalised to females and future work should replicate this study to determine if it can be generalised to the female athlete. The recruitment of only adult males from a single site suggests interpretation of these results with caution in other populations. We did not measure quadriceps strength in the control group. However, it is assumed that quadriceps strength was symmetrical (>90% LSI).^65^ We were unable to assess potential differences between patients with different graft types due to the small sample size. The foot was treated as rigid-body segment and this might have overestimated the ankle work calculations,^66^ however, an identical method was applied to all participants’ data, thus not affecting the between limbs and groups comparisons. Although musculoskeletal modelling allows estimating in vivo muscle forces without invasive methods, it is not without limitations. Since measuring muscle forces in vivo during jumping is not feasible, we do not have a direct test for the accuracy of the computed muscle forces. By using EMG activation patterns to constrain the muscle force estimations, we limited the error in predicting the timing of activations. Additionally, we used a generic model and not a subject-specific modelling approach that incorporates each subject’s lower limb anatomy. Finally, a cross-sectional design was used, which provides only a single time point to evaluate biomechanical differences after ACLR. Future research is necessary to determine the clinical utility of vertical jump performance metrics in athletes after ACLR and association with athlete’s ability to RTS as well as predictive validity for knee reinjury.

## Conclusion

During vertical jumps, male athletes after ACLR at the time to return to sport still exhibit knee biomechanical deficits, despite having achieved symmetry in horizontal hop distance and strength tests. The vertical jump performance metrics like jump height and reactive strength index can better identify interlimb differences than the commonly used hop distance. Thus, in the absence of more advanced biomechanical equipment, vertical jump testing may be a better clinical option for return to sport decision making.

## Data Availability

Data are available on reasonable request.
