# Supplementary material for: Single leg vertical jump performance identifies knee function deficits at return to sport after ACL reconstruction in male athletes
Source: Br J Sports Med. 2022 Feb 8;56(9):490–8. doi: 10.1136/bjsports-2021-104692 (PMC9016240; doi:10.1136/bjsports-2021-104692)
Supplement: Supplementary data [file bjsports-2021-104692supp001.pdf]

Supplementary file 1. Joint work percentage contribution during the single leg vertical jump and the single leg drop jump

				Involved-Uninvolved		Involved-Controls		Uninvolved-Controls	
	**Involved**	**Uninvolved**	**Controls**	**p value**	**d**	**p value**	**d**	**p value**	**d**
**Single leg vertical jump**									
**Propulsion**									
**Work contribution%**									
Hip	35.97±8.52	30.13±7.42	32.49±5.35	**<0.001**	**0.73**	0.24		0.51	
Knee	28.81±7.57	34.20±6.52	33.85±6.54	**0.001**	**0.78**	**0.043**	**0.77**	0.98	
Ankle	35.22±7.15	35.68±5.34	33.65±5.09	0.90		0.73		0.57	
**Landing**									
**Work contribution%**									
Hip	38.58±13.92	33.63±12.0	30.24±9.62	**0.045**	**0.38**	0.05		0.60	
Knee	29.47±9.33	32.97±8.13	34.45±9.98	0.16		0.17		0.85	
Ankle	31.95±13.78	33.40±12.48	35.31±13.58	0.67		0.63		0.84	
**Single leg drop jump**									
**Absorption**									
**Work contribution%**									
Hip	33.59±8.18	24.57±6.86	24.78±7.49	**<0.001**	**1.19**	**<0.001**	**1.18**	0.99	
Knee	32.98±7.68	38.92±5.43	35.28±9.50	**0.004**	**0.89**	0.58		0.26	
Ankle	33.43±7.99	36.51±8.16	39.94±11.39	0.16		0.07		0.45	
**Generation**									
**Work contribution%**									
Hip	30.89±6.04	21.42±6.73	22.80±5.22	**<0.001**	**1.48**	**<0.001**	**1.55**	0.86	
Knee	29.65±6.17	38.52±4.83	35.94±5.49	**<0.001**	**1.60**	**0.001**	**1.15**	0.26	
Ankle	39.45±5.70	40.05±4.80	41.26±6.68	0.73		0.56		0.77	

For the ACLR athletes, data are presented for both the reconstructed ('involved') and contralateral ('uninvolved') leg along with one (randomly selected) leg of the control subjects ('Controls') as mean±SD. The between-group comparisons show p values, and effect sizes (d) only where p<.05. **Bold** indicates statistically significant differences and their respective effect sizes.
